# EFFICACY OF LOW-LEVEL LASER THERAPY ON FISTULA-IN-ANO TREATMENT

**DOI:** 10.1590/0102-672020210001e1572

**Published:** 2021-05-14

**Authors:** Carlos Henrique Marques dos SANTOS, Felipe dos Santos GUIMARÃES, Fernanda Helene Reis BARROS, Guilherme Apolinário Laureano LEME, Lucas Dutra Madrid da SILVA, Sandro Endrick de Oliveira SANTOS

**Affiliations:** 1 Coloproctology Department, University Hospital Maria Aparecida Pedrossian, Federal University of Mato Grosso do Sul, Campo Grande, MS, Brazil; 2 Anhanguera-Uniderp University, Campo Grande, MS, Brazil

**Keywords:** Anal fistula, Light therapy of low intensity, Healing, Therapy, Inflammation, Fístula retal, Terapia com luz de baixa intensidade, Cicatrização, Tratamento, Inflamação

## Abstract

**Background:**

Treating anal fistulae is still a great challenge due to the possibility of fecal incontinence after surgery and that the use of laser has been gaining space in medicine, including as an inducing method of healing.

**Aim:**

To evaluate the efficacy of low-level laser therapy on fistula-in-ano treatment in rats.

**Methods:**

Fifteen male Wistar rats weighing approximately 250-300g were used, which were subjected to the anal fistula induction procedure and after 30 days were distributed into two groups: control group (CG, n=5) and laser group (LG, n=10) observed for another 30 days. In the CG no treatment was performed and, in the LG, low-level laser therapy was applied in fistulous tracts daily. The closure of the fistulous tract, the area of the remaining tract, the inflammatory infiltrate and vascular congestion were evaluated.

**Results:**

There was no complete closure of the tract in any of the animals. The mean area of the remaining tract was 847.2 µm^2^ in the CG and 248.5 µm^2^ in the LG (p=0.001). The mean inflammatory infiltrate score was 2.4 in the CG and 1.3 in the LG (p=0.0285), while in the evaluation of vascular congestion, 1.6 was observed in the CG and 0.6 in the LG (p=0.031).

**Conclusions:**

Low-level laser therapy was able to reduce the area of the fistulous tracts as well as decrease the inflammatory process and local vascular congestion.

## Graphical abstract

[Fig f1a]

**Figure f1a:**
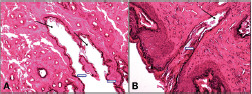
Fistulous area being A control and B laser group: Arrows indicate fistulous tract much thinner in B

[Fig f1as]

**Figure f1as:** Área fistulosa sendo o grupo de controle A e laser B: as setas indicam o trato fistuloso muito mais fino em B

## RESUMO

**Racional:**

Tratamento de fístulas anais ainda é grande desafio devido à possibilidade de incontinência fecal pós-operatória; o uso do laser vem ganhando espaço na medicina, inclusive como método indutor de cicatrização.

**Objetivo:**

Avaliar a eficácia da terapia a laser de baixa potência no tratamento de fístula perianal em ratos.

**Métodos:**

Foram utilizados 15 ratos Wistar machos com peso aproximado de 250-300 g, os quais foram submetidos à indução da fístula anal e após 30 dias foram distribuídos em dois grupos: grupo controle (GC, n=5) e grupo laser (GL, n=10) observado por mais 30 dias. No GC nenhum tratamento foi realizado e no LG laserterapia de baixa intensidade foi aplicada nos trajetos fistulosos diariamente. Foram avaliados o fechamento do trajeto fistuloso, a área do trajeto remanescente, o infiltrado inflamatório e a congestão vascular.

**Resultados:**

Não houve fechamento completo do trajeto fistuloso em nenhum dos animais. A área média do trajeto remanescente foi de 847,2µm^2^ no GC e 248,5µm^2^ no GL (p=0,001). O escore médio do infiltrado inflamatório foi de 2,4 no GC e 1,3 no GL (p=0,0285), enquanto na avaliação da congestão vascular foi observado 1,6 no GC e 0,6 no GL (p=0,031).

**Conclusões:**

A terapia a laser de baixa potência foi capaz de reduzir a área dos trajetos fistulosos, bem como diminuir o processo inflamatório e a congestão vascular local.

## INTRODUCTION

The perianal fistula is a canal formed by an internal opening in the anus and the perianal skin, forming a tract of fibrosis epithelized and/or filled with granulation tissue, almost always resulting from an cryptoglandular abscess^10^ ^,^ ^14^.

Depending on the spread of the abscess in relation to the anal and rectal spaces, the resulting fistula may have paths involving varying amounts of the anal sphincter. Thus, the treatment with the best cure rate for anal fistulas - fistulotomy - which consists of opening and curettage of the fistulous tract, cannot be applied in all cases, because the greater the number of sectioned muscle fibers, the greater the risk of fecal incontinence^12^ ^,^ ^17^.

Therefore, transsphincteric fistulas, which are the most frequent after interphincteric fistulas, are still challenging today because they need a treatment capable of curing them without causing fecal incontinence, and there is still no standardized technique for this situation, which is an open field to research.

Since its beginning, the laser has found application in medicine, especially in the surgical field. Most applications are based on photothermal and photoablative interactions of the laser with the tissues and are often used to cut, join and even destroy certain tissues. The use of laser in clinical applications is based on the potential for non-thermal interactions with the tissue, which would be able to modulate certain biological processes, in particular tissue regeneration process^16^.

Low-level laser therapy would act on the chromophores present in the mitochondrial membrane, resulting from an increase in the synthesis of adenosine triphosphate (ATP) and, consequently, an increase in the levels of reactive oxygen species, which in turn would act as intracellular signals capable of regulating the activity of various enzymes, such as phosphatases and kinases, and to promote gene transcription. This, in turn, promotes protein synthesis that culminates in increased cell proliferation and migration, modulation of cytokines, growth factors, inflammatory mediators and tissue oxygenation, which in theory would be beneficial in the treatment of anal fistulas^5^ ^,^ ^8^. 

Thus, the objective of the present research was to evaluate the efficacy of low-level laser in the treatment of perianal fistulas in rats.

## METHODS

The study was approved by the Anhanguera-Uniderp University Ethics Committee on Animal Use and all standards established by the Brazilian College of Animal Experimentation were followed.

Fifteen male, adult and albino Wistar rats, weighing approximately 250-300 g each were used, which received water and feed ad libitum during the study.

The animals were anesthetized peritoneally to create anal fistulas using xylazine hydrochloride 2%, at a dose of 10 mg/kg, and ketamine hydrochloride 10%, at a dose of 50 mg/kg, in a proportion of 2:1, using 0.1ml of the solution for every 100 g of weight. 

**FIGURE 1:**
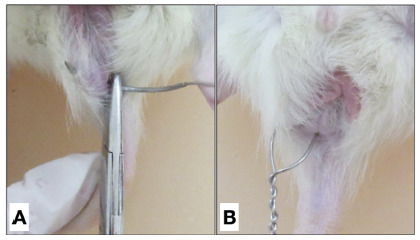
A) Transection of the anal sphincter with steel wire; B) steel wire positioned for creating the anal fistula

After anesthetization, anal fistulas were created by steel wire number 0 (Aciflex^®^), transfixing the anal sphincter, with the needle being introduced into the pectinous line in the right lateral position and externalized approximately 1 cm laterally to the right anal margin (Figure 1A). The steel wire was cut and twisted (Figure 1B), being left for 30 days^2^.

After 30 days, new anesthesia was performed and the steel wire was removed, and the animals were then distributed into two groups: control group (CG) with removal of the steel wire and observation for 30 days without any treatment (n=5); and laser group (LG) with removal of the steel wire and daily application of low-level laser for 30 days (n=10)

In the animals of the LG 4J of red laser was applied to the external orifice of the fistula, in the anus of the animal and between these points, using the following configurations: model Foton Laser III, power of 100 mw, beam area of 0.028 cm^2^, density of energy of 4J/cm^2^, power density (DP) of 3.57W/cm^2^, application time of 1.12 s per point, visible emitter 660 nm wavelength red laser, InGaAlP active medium (indium + gallium + aluminum + phosphorus). The formulas used were as follows:

$$D\;\left(J/cm^{2}\right)=P\;x\;T/1000\;x\;A$$ (1)

E=PxT/1000 (2)

T=DxAF/P (3)

Where equation 1, represents the dosimetric formula, where the letter D represents the power density in Joules, the letter P is equal to the power in mW (microwatts), the letter T represents the time in seconds and the letter A the area in square centimeters; equation 2, represents the energy formula, where the letter E is equal to the energy in Joules, the letter P is the power in mW and the letter T is the time in seconds; equation 3, in turn represents the calculation of the time of exposure of the tissue to the light beam, where the letter D represents the energy density in Joules, the letters AF the area of the light beam, the letter P the power in mW and the letter T the time in seconds. 

After 30 days, under anesthesia (similar to the previous procedure), all animals were euthanized with deepening of the anesthetic plan. Subsequently, the specimens were removed for histological slides. Perianal trichotomy, incision with a cold scalpel, removal of a cube involving the anal canal and the entire fistulous tract was performed. The specimens were stored in tubes identified with 10% formaldehyde, until the histological slides were prepared.

The slides were stained with H&E for histological analysis. The variables analyzed were: closure of the lumen of the fistulous tract; area of the remaining fistulous tract; intensity of the inflammatory process; and vascular congestion.

Persistence or closure of the fistulous tract were analyzed through: visualization by microscopy the persistence of the fistula; closure considered only when the entire tract was closed; maintenance, even if the tract was short, was considered persistence.

Area of the remaining fistulous tract was measured: under optical microscopy, in a coronal section of the anal canal, in which the area in pixels of the remaining fistulous tract in its entire length was measured and converted into square micrometers (µm^2^) after marking the entire wall of the fistulous tract with a cursor.

To determine the inflammatory infiltrate, scores were applied according to the count of the inflammatory foci. When no inflammatory focus was observed, score 0 (absent), one to two foci, score 1 (mild), three to four foci, score 2 (moderate), more than four foci, score 3 (intense) were determined.

Vascular congestion was classified as: 0 - absent; 1 - mild; 2 - moderate; and 3 - intense.

### Statistical analysis

The results were subjected to statistical treatment by Kruskall-Wallis and Mann-Whitney non-parametric tests, with a significance level of 5%. Data analysis was performed using the Statistical Package for the Social Sciences 24 (SPSS 24).

## RESULTS

There was no complete closure of the fistulous tracts in any animal. The average area of the remaining fistulous tracts was 847.2 µm^2^ in the CG and 246.5 µm^2^ in the LG (p=0.001,Table 1, Figure 1).

**TABLE 1:** Evaluation of the area of the remaining fistulous tracts of the studied groups according to the histological analysis (values in square micrometers)

Groups		
Rats	Control	Laser
1	946	354
2	501	214
3	894	245
4	782	278
5	1113	261
6	-	219
7	-	115
8	-	286
9	-	260
10	-	233
Average	847.2	246.5*

*p=0,001

**FIGURE 2:**
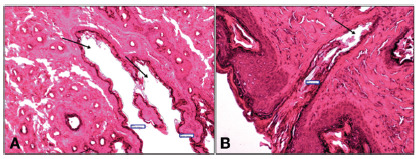
Photomicrograph showing the measurement of the area of the fistulous tract after treatment: A) CG; B) LG. Arrows indicate the patent fistulous tract (H&E 400X)

The inflammatory process was evaluated in the animals of both groups, with a higher average in the control group than in those treated with laser, 2.4 versus 1.3 (p=0.00285, Table 2).

**TABLE 2:** Evaluation of the degree of inflammation in the region assessed according to groups

Groups		
Rats	Control	Laser
1	2	1
2	2	1
3	3	2
4	2	1
5	3	3
6	-	2
7	-	1
8	-	0
9	-	1
10	-	1
Average	2.4	1.3*

*p=0,0285

The mean scores of vascular congestion were 1.6 in the CG and 0.6 in the LG (p=0.031), as can be seen in Table 3.

**TABLE 3:** Evaluation of the degree of vascular congestion in the studied region according to the groups

Groups		
Rat	Control	Laser
1	0	1
2	1	1
3	3	1
4	1	0
5	3	1
6	-	0
7	-	1
8	-	0
9	-	0
10	-	1
Average	1.6	0.6*

*p=0,031

## DISCUSSION

The purpose of any treatment used for perianal fistulas is to bring about a complete cure of the fistulous tract without causing fecal incontinence. In the model used here, no fistula was closed in any of the animals. However, it was observed that in the group treated with low-level laser there was a significant reduction in the lumen of the tracts, which allows us to infer that perhaps with a longer period of treatment there could be total closure.

Terzi et al.^16^ evaluated the effect of low-level laser in patients with anal fistula and achieved complete closure in 40% of cases, in addition to a reduction in purulent drainage in 19% of those who did not have closure. A partial benefit was also observed in the present research, although it is not possible to make a direct comparison because it is an experimental study. In addition, there is a fundamental difference in the method, as Terzi et al.^16^ closed the internal orifice and applied the laser inside the tracts, differently from what was practiced here, with external application of therapy.

Also, Wilhelm et al.^20^ achieved complete closure in 64.1% of the fistulas in a study with 177 laser-treated patients, reaching 85% when those who failed underwent a second treatment, demonstrating that in addition to the excellent result, there is no contraindication for prolonged treatment. These authors, however, added the closure of the internal orifice of the fistula, which was not done in the present study.

Similar results were obtained by Giamundo et al.^7^ who observed 71% of healing of anal fistulas, also with the internal application, however, they concluded that the best results were obtained in those who used a loose seton previously.

These studies have in common the use of the same equipment and technique^7^ ^,^ ^16^ ^,29^ - FiLaC (Fistula-tract Laser Closure) - also achieving very similar results. This laser technique aims to remove the epithelium and granulation tissue from the inside of the fistulous tracts, stimulating the epithelium healing ^7^ ^,^ ^20^. There is, however, no experimental research like the one presented here that uses another way of applying the low-level laser so that we could make direct comparisons. When obtaining a satisfactory result with the external application of the low-level laser as in the present research, we believe that this would be a superior option, because in addition to not causing pain there is also no need for a probe for internal application and, therefore, would allow the outpatient use, dispensing inpatients and anesthesia.

Considering that the anal fistula is the chronic phase of the abscess^12^, that is, it persists with local inflammation, the reduction of the inflammatory process in the group under laser treatment allows us to say that these animals were closer to healing than those in the control group, reinforcing the hypothesis that longer therapy could be fully effective.

This anti-inflammatory potential of laser has already been demonstrated in some publications, such as by Uslu et al.^18^ who observed that laser therapy was able to reduce the inflammatory process in induced periodontitis in rats. Requena et al.^13^ verified in an experimental model of pig skin that in the photodynamic therapy with pulsed light, with histological evaluation, there was a small change in the collagen fibers and reduction of the inflammatory process, confirming the observed here regarding the reduction of inflammation with the use low-level laser therapy.

In the present study, a significant reduction in vascular congestion was also observed, which contributes to confirming the reduction of the inflammatory process, since this event is one of the first stages of the inflammation, that is, the lower the degree of vascular congestion, the lower should be the inflammation. Although this experimental research does not allow us to state with total confidence, it can be inferred that in clinical situations the reduction of inflammation would lead to improvement of symptoms, even without complete closure of the fistula. According to Walsh et al.^19^, low-frequency laser can induce immediate reduction in the isometric tension of vascular smooth muscle, and its relaxation can contribute to analgesic effects and decrease in vascular congestion, a decrease that is also favored by stimulating neovascularization.

The low-level laser applied externally can promote healing because it decreases the inflammatory response, promotes angiogenesis, fibroplasia, collagen synthesis, ATP synthesis and cell matrix differentiation^6^. These effects already observed in previous publications are necessary for the healing of the fistulous tract and may explain the mechanism by which there was a reduction in the lumen of the fistulous tract and the inflammatory process in the animals studied here.

Many publications^1^ ^,^ ^3^ ^,^ ^4^ ^,^ ^7^ ^,^ ^11^ ^,^ ^16^ ^,^ ^19^ ^,^ ^20^ have appeared in recent years on the use of low-level laser in the treatment of anal fistula, but all in humans and with a specific product that requires a probe for internal laser application. Despite the good results presented, as also seen in other techniques^9^ ^,^ ^15^, it is necessary to consider the high cost, the need of hospitalization and the need to close the internal orifice according to the description of the technique. However, there is no experimental research that, like the one presented here, has tested another route of application, potentially less painful and less costly, as it is simpler. 

It can be considered that this is a limiting factor of our research, the lack of data in the literature to compare the results. On the other hand, although there was no complete closure of any of the fistulous tracts, its significant area reduction and reduction of the inflammatory process leads us to believe that the treatment carried out for a longer time could reach the complete response and should be the objective of future research.

## CONCLUSION

Low-level laser therapy was not able to lead to complete closure of the fistulous tracts in the model used, but it promoted partial closure, decreased inflammatory process and vascular congestion around the fistulas.
